# The Cytochrome *bd* Complex Is Essential for Chromate and Sulfide Resistance and Is Regulated by a GbsR-Type Regulator, CydE, in *Alishewanella* Sp. WH16-1

**DOI:** 10.3389/fmicb.2018.01849

**Published:** 2018-08-10

**Authors:** Xian Xia, Shijuan Wu, Liqiong Li, Biao Xu, Gejiao Wang

**Affiliations:** State Key Laboratory of Agricultural Microbiology, College of Life Sciences and Technology, Huazhong Agricultural University, Wuhan, China

**Keywords:** chromate resistance, sulfate reducing, cytochrome *bd*, *Alishewanella*, CydE

## Abstract

Sulfate-reducing bacteria are a group of microorganisms that use sulfate as an electron acceptor. These bacteria are useful in the bioremediation of heavy metal pollution since they can reduce/precipitate metals. Previously, we identified the *Alishewanella* strain WH16-1 from soil of a copper and iron mine and determined that it can reduce sulfate and chromate and that it was tolerant to many heavy metals. In this study, we investigated the chromate reduction mechanism of strain WH16-1 through Tn*5* transposon mutagenesis. A cytochrome *bd* (*cytbd*) Tn*5* mutant was generated (Δ*cytbd*), and a detail analysis showed that the following: (1) gene *cydE* (coding for a GbsR-type regulator) was co-transcribed with the two subunits coding genes of the Cytochrome *bd* complex (Cytbd), namely, *cydA* and *cydB*, based on RT-PCR analysis, and similar gene arrangements were also found in other *Alteromonadaceae* family strains; (2) the chromate resistance level was dramatically decreased and chromate reduction efficiency also decreased in strain Δ*cytbd* compared to the wild-type and a complemented strain (Δ*cytbd*-C); (3) Cytbd could catalyze the decomposition of H_2_O_2_ according to the analyses of H_2_O_2_ decomposition ability, cellular H_2_O_2_ contents, H_2_O_2_ inhibition zone, and H_2_O_2_ sensitivity tests; (4) surprisingly, chromate was not an inducer of the expression of Cytbd, but sulfate induced expression of Cytbd, and sulfate/sulfide resistance levels were also decreased in the Δ*cytbd* strain; (5) the addition of sulfate enhanced the chromate resistance level and reduction efficiency; (6) Cytbd expression was repressed by CydE and derepressed by sulfate based on an *in vivo* bacterial one hybrid system and *in vitro* EMSA tests; and (7) DNA footprinting and short-fragment EMSA tests revealed two binding sites of CydE in its promoter region. All these results showed that Cytbd is negatively regulated by CydE and derepressed by sulfate. In addition, Cytbd contributes to the resistance of sulfate and sulfide, and sulfide could be used as a reductant to reduce chromate. Moreover, Cytbd is essential to decompose H_2_O_2_ to decrease cellular oxidative stress. Thus, the regulation and function of Cytbd may explain why sulfate could enhance chromate reduction.

## Introduction

Sulfate-reducing bacteria (SRB) are a diverse group of prokaryotes that use sulfate as the terminal electron acceptor and produce H_2_S (Muyzer and Stams, 2008). They are widely distributed and play a key role in the environment (Muyzer and Stams, 2008; Barton and Fauque, 2009). Various SRB have exhibited great potential for environmental bioremediation applications, such as participating in the precipitation of heavy metals to produce metal sulfides (Xia et al., 2016; Zhou et al., 2016), reduction of toxic metals (Barton and Fauque, 2009), degradation of azo dyes (Pandey et al., 2007), and nitroaromatic compound respiration (Barton and Fauque, 2009).

Chromate [Cr(VI)] is highly soluble and can easily cross cellular membranes. Once inside the cell, chromate exhibits a variety of toxic, mutagenic, and carcinogenic effects since it induces reactive oxidative species and affects both DNA and protein functions (O’Brien, 2003; Sobol and Schiestl, 2012). However, its reduction product, Cr(III), is insoluble and has low toxicity (Dhal et al., 2013; Viti et al., 2014). In addition to Cr(VI) reduction, other bacterial Cr(VI) detoxification mechanisms have been found, such as efflux (Viti et al., 2014), reduction of cellular oxidative stress (Ramírez-Díaz et al., 2007; Branco et al., 2008), and DNA repair (Ramírez-Díaz et al., 2007; Viti et al., 2014). In addition, sulfur metabolism is found to be relevant to Cr(VI) detoxification in many bacteria (Cheung and Gu, 2007; Ramírez-Díaz et al., 2007; Thatoi et al., 2014; Viti et al., 2014; Joutey et al., 2015).

Chromate is chemically analogous to sulfate and enters cells mediated by the sulfate ABC transporter CysPUWA in various bacteria (Viti et al., 2014). Some SRB are also chromate-reducing bacteria (CRB) (Viti et al., 2014; Xia et al., 2016). Accordingly, Cr(VI) induces the expression of the sulfate transporter and competes with sulfate in some bacteria (Viti et al., 2014). Moreover, the products of sulfur assimilation are involved in Cr(VI) detoxification. H_2_S, cysteine, and glutathione (GSH) are capable of directly reducing Cr(VI) (Cheung and Gu, 2007; Thatoi et al., 2014; Joutey et al., 2015). In addition, GSH also plays an important role in maintaining cellular sulfhydryl groups in their reduced form when exposed to oxidative stress induced by Cr(VI) (Presnell et al., 2013; Viti et al., 2014). However, many details concerning the effects of sulfur metabolism on Cr(VI) detoxification remain unclear.

Cytbd is a terminal respiratory oxidase found in many prokaryotes and is composed of two subunits, CydA and CydB (Giuffre et al., 2014). CydC and CydD are also needed for the assembly of Cytbd in *Escherichia coli* (Borisov et al., 2011), while CydX is also essential for the activity of Cytbd in some bacteria (Sun et al., 2012; VanOrsdel et al., 2013; Chen H. et al., 2015). Cytbd is involved in energy supply, bacterial virulence, and resistance to oxidative and nitrosative stresses (Borisov et al., 2013; Giuffre et al., 2014; Roop et al., 2015). Recently, Cytbd was also found to be associated with sulfide resistance in *E. coli*, since sulfide could inactivate heme–copper family respiratory oxygen reductases (cytochrome *bo*_3_) but not the copper-free Cytbd (Forte et al., 2016; Korshunov et al., 2016). In addition, the expression of Cytbd is regulated by the transcriptional regulators Arc, Fnr, and CydR, depending on environmental oxygen concentrations in *E*. *coli* and *Azotobacter vinelandii* (Wu et al., 1997; Borisov et al., 2011). Potential regulator genes containing helix-turn-helix (HTH) conserved domain sequences were observed adjacent to the *cytbd* operon in some bacteria (Degli Esposti et al., 2015). However, these potential regulators, such as the GbsR type (Nau-Wagner et al., 2012; Lee et al., 2013), have not been reported to regulate the expression of Cytbd. Furthermore, no study concerning the relevance between Cytbd and chromate resistance has been reported thus far.

*Alishewanella* sp. WH16-1 (=CCTCC M201507) was isolated from soil of a copper and iron mine. It possesses great potential in metal bioremediation since it reduces sulfate and chromate or generates CdS/PbS precipitation (Xia et al., 2016, 2018; Zhou et al., 2016). Strain WH16-1 also showed a high tolerance to Cr(VI) (MIC of 45 mM) (Zhou et al., 2016). However, its Cr(VI) resistance and reduction mechanisms remain to be explored. A first step of this study was to investigate the Cr(VI) detoxification mechanism of strain WH16-1 through Tn*5* transposon mutagenesis. Later, we found that Cytbd was relevant to chromate, sulfate, and sulfide resistance. Interestingly, the transcription of *cytbd* was repressed by a GbsR-type regulator (named CydE) and depressed by sulfate.

## Materials and Methods

### Bacterial Strains, Plasmids, and Growth Conditions

The bacterial strains and plasmids used in this study are listed in **Supplementary Table S1**, and the primers are listed in **Supplementary Table S2**. *Alishewanella* sp. WH16-1, *E. coli*, and their derivative strains were cultured at 37°C in Luria-Bertani (LB) medium unless otherwise noted. Stock solutions of rifampin (Rif, 50 mg mL^−1^), kanamycin (Km, 50 mg mL^−1^), chloramphenicol (Cm, 25 mg mL^−1^), tetracycline (Tet, 5 mg mL^−1^), K_2_CrO_4_ (1 M), Na_2_SO_4_ (1 M), and Na_2_S (0.1 M) were added when required.

### Transposon Mutagenesis and Construction of a Complemented Strain

To identify the molecular mechanism of Cr(VI) detoxification of strain WH16-1, Tn*5* transposon mutagenesis was used for screening Cr(VI) resistance genes. Transposon insertion mutants were generated with a suicide plasmid pRL27 (Larsen et al., 2002) transferred from the donor strain *E. coli* S17-1 to the recipient strain WH16-1 using the filter mating method (Smith and Guild, 1980). After conjugation, the Tn*5* (Km^r^) transposon was randomly inserted into the chromosome DNA of strain WH16-1, generating a library of insertion mutants. Selection was done on LB plates with Rif (50 μg/mL) and Km (50 μg/mL) to obtain strains in which transposition had occurred. The transconjugants were then plated on two LB plates with or without 20 mM K_2_CrO_4_, and the colonies that were unable to grow in the presence of K_2_CrO_4_ were reserved and subjected to further analyses. Cloning of genes neighboring the Tn*5* transposon was performed according to the plasmid rescue method described before (Chen F. et al., 2015). The resulting neighboring sequences were searched against the whole genome of strain WH16-1 (Xia et al., 2016) using the NCBI BLAST server.

To identify the function of Cytbd, a complemented strain was constructed. The whole *cytbd* operon was cloned into the pCT-Zori plasmid using *Sac*I and *Hind*III restriction enzyme sites. The generated plasmid was transferred into the mutant strain Δ*cytbd* by conjugation from *E. coli* S17-1 to obtain a complemented strain, Δ*cytbd*-C.

### Analysis of *cytbd* Operon and Co-transcription

For analysis of *cytbd* conservation, homologous operon sequences from members of the *Alteromonadaceae* were selected from their genomes. They were *Alteromonas macleodii* HOT1A3^T^ (NZ_CP012202), *Alteromonas marina* AD001^T^ (NZ_JWLW01000010), *Alteromonas* sp. Mex14 (CP018023), *Alteromonas* sp. Nap 26 (LSMP01000036), *Alteromonas australica* H17^T^ (NZ_CP008849), *Salinimonas chungwhensis* DSM 16280^T^ (NZ_KB899391), *Glaciecola pallidula* DSM 14239^T^ (NZ_AUAV01000023), *Alishewanella agri* BL06^T^ (AKKU01000001), *Alishewanella jeotgali* KCTC 22429^T^ (AHTH01000001), *Paraglaciecola arctica* BSs20135^T^ (NZ_BAEO01000055), and *Lacimicrobium alkaliphilum* YelD216^T^ (NZ_CP013650). Phylogenetic analysis was carried out based on the *cytbd* operon (GbsR family regulator CydE, CydA, and CydB) amino acid sequences. The analysis was performed by MEGA 6.0 (Tamura et al., 2013) with a neighbor joining algorithm, and 1,000 bootstrap repetitions were computed to estimate the reliability of the tree. In addition, the operon arrangement in these strains was also analyzed.

For co-transcription analysis, strain WH16-1 was incubated to an OD_600_ of approximately 0.3 in 100 mL LB broth, followed by incubation with 1 mM K_2_CrO_4_ for 3 h. Total RNA was extracted by Trizol reagent (Invitrogen), and DNA was removed by digestion with DNase I (Takara). Reverse transcription was conducted with a RevertAid First Strand cDNA Synthesis Kit (Thermo) with 300 ng total RNA for each sample. The resulting cDNA was used as a template to amplify the fragments between genes in the *cytbd* operon. Genomic DNA was used as a positive control. The total RNAs of strain WH16-1 and ddH_2_O were used as negative controls. Primers are shown in **Supplementary Table S2**.

### Reporter Gene Construction

The putative promoter and promoter-*cydE* regions (**Supplementary Figure S1A**) were each PCR amplified from genomic DNA of strain WH16-1. Each DNA fragment was then cloned into plasmid pLSP-kt2*lacZ* using *Eco*RI–*Bam*HI restriction enzyme sites. The resulting constructs were designated as pLSP*-*promoter-*lacZ* (**Supplementary Figure S1B**) and pLSP-promoter-*cydE-lacZ* (**Supplementary Figure S1C**). *E. coli* DH5α containing pLSP*-*promoter-*lacZ* or pLSP-promoter-*cydE-lacZ* was incubated in LB medium. Overnight cultures were diluted 100 times with fresh medium and incubated for approximately 4 h (OD_600_ approximately 0.3). Next, Na_2_SO_4_ (0, 5, 25, and 50 mM) and K_2_CrO_4_ (0, 1, and 5 mM) were added to the cultures. The cultures were then distributed into tubes after a 6-h incubation. β-Galactosidase enzymatic assays were performed using the method described by Li et al. (2015).

### Chromate/Sulfate/Sulfide Sensitivity and Chromate Reduction Assay

Strains WH16-1, Δ*cytbd*, and Δ*cytbd*-C were each inoculated into 5 mL LB and incubated at 37°C with shaking at 150 rpm. When the OD_600_ reached approximately 0.8–1.0, the strains were each inoculated into 100 mL LB with the presence of 500 mM Na_2_SO_4_, 200 μM Na_2_S, 3 mM K_2_CrO_4_, or no addition. Na_2_SO_4_ powder was added to LB medium before sterilization, while K_2_CrO_4_ and Na_2_S were added from stock solutions. In addition, LB plates with 0 or 3 mM K_2_CrO_4_ were used for Cr(VI) sensitivity analysis. For observing Cr(VI) reduction, strains were incubated in LB broth with 1 mM K_2_CrO_4_. To maintain consistent growth conditions, K_2_CrO_4_ was added when OD_600_ reached 0.6. At designated times, culture samples were taken for measuring OD_600_ and chromate amounts by spectrophotometry (DU800, Beckman) and atomic absorption spectrometry (AAS; 986A, Beijing Puxi General Instrument Co., Beijing, China), respectively.

### Effects of Cytbd on Cellular Oxidative Stress

To analyze the effects of Cytbd on oxidative stress, membrane proteins of strains WH16-1, Δ*cytbd*, and Δ*cytbd*-C were extracted to react with H_2_O_2_ and chromate. The membrane protein extraction was performed as described previously by Das et al. (2005), and the protein concentration was determined by the Lowry method (Lowry et al., 1951). Then, 10 mg/L of membrane proteins was reacted with 10 mM hydroquinone and 10 mM H_2_O_2_ or K_2_CrO_4_ in Tris–HCl (pH 8.5) buffer for 30 min under anoxic conditions in a N_2_ chamber. The residual H_2_O_2_ was measured by the Amplex red/horseradish peroxidase assay (Mishin et al., 2010), and chromate concentrations were determined as mentioned above.

To gain more insight into the effects of Cytbd on cellular oxidative stress, cellular H_2_O_2_ contents of the WH16-1, Δ*cytbd*, and Δ*cytbd*-C strains were determined. The strains were incubated to an OD_600_ of approximately 0.3 in 100 mL LB. K_2_CrO_4_ was then added to the cultures until the final concentrations reached 1 mM. Cells were centrifuged and washed twice with potassium phosphate buffer (50 mM, pH 7.7). The pellets were lysed via sonication on ice for 3 min and centrifuged for 5 min at 12,000 rpm to remove particulate materials. H_2_O_2_ amounts were measured as mentioned above.

Moreover, inhibition zone and H_2_O_2_ sensitivity tests were performed. For the inhibition zone test, cultures of each strain (OD_600_ approximately 0.8–1.0) were added to LB agar medium, and 200 μL of 3% H_2_O_2_ was added to the Oxford cup (Wang et al., 2009). For the H_2_O_2_ sensitivity assay, 5 μL overnight cultures of WH16-1, Δ*cytbd*, and Δ*cytbd*-C (OD_600_ 0.8–1.0) were dropwise added onto LB agar media containing various amounts of H_2_O_2_ (0, 0.05, 0.1, 0.5, and 1 mM).

### Effects of Sulfate on Chromate Reduction and Resistance

Chemically defined medium (CDM) was selected to test the effects of sulfate on Cr(VI) reduction and resistance in strain WH16-1. The components of the CDM medium were the same as previously described (Weeger et al., 1999), except for replacing sodium lactate, magnesium sulfate and sodium sulfate with maltose, magnesium, and sodium chloride, respectively. This medium contained 0.12 mM SO_4_^−2^ and no other forms of sulfur. Strain WH16-1 was incubated with or without 100 μM Cr(VI) and additional 0, 5, or 10 mM sulfate in CDM medium. The remaining Cr(VI) in the medium was determined as mentioned above.

### Bacterial One-Hybrid System Assay

The DNA binding activity of CydE was tested *in vivo* with a bacterial one-hybrid system (Guo et al., 2009). The *cydE* coding sequence was amplified and cloned into the pTRG vector using *Bam*HI–*Eco*RI restriction enzyme sites to obtain a plasmid pTRG-*cydE*. The promoter sequence of the *cydE* (**Supplementary Figure S1A**) was amplified and inserted directly into *Xcm*I site of pBXcmT, yielding the pBX-promoter plasmid. The next steps were followed as previously described (Guo et al., 2009; Shi et al., 2017). The pTRG-*cydE* and pBX-promoter plasmids were co-transformed into *E. coli* XL1-Blue and grew on selective screening medium plates (Guo et al., 2009). In addition, *E. coli* XL1-Blue containing the pBX-MthspXp and pTRG-Rv3133c plasmids served as the positive controls, while *E. coli* XL1-Blue containing the empty vectors pBXcmT or pTRG was used as negative controls (Guo et al., 2009; Shi et al., 2017).

### Cloning, Expression, and Purification of CydE

The CydE coding sequence was also amplified from DNA of strain WH16-1 using specific primers (**Supplementary Table S2**) that were designed to contain the restriction sites for *Bam*HI and *Hind*III. The PCR product was digested with these enzymes and cloned into pET28a generating plasmid pET28a-*cydE*. After DNA sequencing confirmation, the plasmid was introduced into *E. coli* BL21 (DE3) cells. CydE was overexpressed by adding 0.1 mM IPTG to cells at an OD_600_ of 0.3–0.4 that were further cultured for 4 h at 28°C. The cells were then harvested by centrifugation (8,000 rpm for 10 min at 4°C). After washing twice with 50 mM Tris–HCl (pH 8.0), the pellets were lysed via French Press at 120 MPa. Next, the soluble supernatant was mixed with 1 mL pre-equilibrated Ni-NTA His Bind Resin (7sea Biotech) and gently agitated at 4°C for 1 h. The resin was transferred into a 10-mL gravity-flow column and washed with 4 mL Tris–HCl with 200 mM imidazole to elute the miscellaneous proteins. The His-tagged CydE protein was eluted in 1 mL Tris–HCl with 500 mM imidazole, and the eluted fractions were analyzed with sodium dodecyl sulfate–polyacrylamide gel electrophoresis (SDS–PAGE). The quality and quantity of the proteins were assessed with spectrophotometry (NanoDrop 2000, Thermo) and SDS–PAGE.

### Electrophoretic Mobility Shift Assay (EMSA)

The DNA probe of *cytbd* promoter sequence (**Supplementary Figure S1**) was generated using the primer pair PromoterF/PrompterR (**Supplementary Table S2**). The PrompterR primer was labeled by fluorophore FAM when needed. In general, DNA binding assay was performed in a 20 μL reaction volume containing 2 μL 10× binding buffer (10 mM Tris, pH 7.5, 10 mM EDTA, 1 mM KCl, 1 mM DTT, 50% glycerol, and 0.1 mg/mL BSA), 100 ng FAM-labeled probe, and different concentrations (0, 0.1, 0.2, and 0.4 μg) of the purified CydE. For competition assay, 0.2, 1, and 2 μg unlabeled probes were added to reaction mixtures containing 0.4 μg CydE and the 100-ng labeled probe. All reaction mixtures were incubated at 37°C for 30 min before being loaded onto an 8% native polyacrylamide gel (Shi et al., 2017). After 1 h of electrophoresis at 120 V in 0.5× TGE buffer (6 mM Tris, 47.5 mM glycine, 0.25 mM EDTA, pH 8.0), gels were exposed to a phosphor imaging system (Fujifilm FLA-5100). For derepression analysis, 0.4 μg CydE was incubated with different concentrations of Na_2_SO_4_ (0, 1, 10, and 100 mM) and K_2_CrO_4_ (1, 10, and 100 mM) for 15 min, and then, 100 ng FAM-labeled probe and other components were added. Gel analysis was carried out as described above.

### DNA Footprinting

One hundred nanograms of FAM-labeled DNA probe was incubated with 0, 0.2, and 0.4 μg CydE, respectively (the reaction system was the same as in EMSA), then digested by 6 × 10^−4^ U/μL DNase I (New England Biolabs) for 10 min at room temperature. Next, the reaction was stopped by addition of 50 mM EDTA and incubation in a water bath at 65°C for 10 min. The digested DNA fragments were purified with a PCR clean-up Gel extraction kit (Macherey-Nagel). Samples were analyzed in a 3730 DNA Analyzer (Applied Biosystems, Foster City, CA, United States), and the electropherograms were aligned with GeneMapper v3.5 (Applied Biosystems, Foster City, CA, United States). For verification of the binding sites, a short-fragment EMSA test was used. The DNA sequences of the two binding sites identified by DNA footprinting were synthesized by Tsingke (Biological Technology Company, Beijing, China). The process of short-fragment EMSA was performed as described above. The final gel was stained by ethidium bromide.

## Results

### Characterization of the Chromate-Sensitive Mutants by Transposon Mutagenesis

Random mutants were generated by mobilization of the suicide plasmid pRL27 from the donor strain *E. coli* S17-1 into the recipient strain WH16-1. Approximately 8,000 Km- and Rif-resistant clones were randomly chosen and initially tested for their ability to grow on LB plates containing 20 mM K_2_CrO_4_. After 48 h of incubation, 40 Cr(VI)-sensitive mutants were obtained. Mutation sites were identified in 13 mutants with decreased Cr(VI) resistance. These mutant genes encoded CydB, ChrB, ferredoxin, iron transporter, DNA repair proteins (UvrC, UvrD, RecA, RecB, RecC, and YebC), and three other proteins (ComEC, ScpA, and a hypothetical protein). The *cydB* mutant (Δ*cytbd*) was selected for this study since it may reveal potentially novel Cr(VI) detoxification mechanisms. The BLAST results showed that the Tn*5* was inserted in the middle of the *cydB* (AAY72_09260) gene.

### The *cytbd* Operon in Strain WH16-1

**FIGURE 1:**
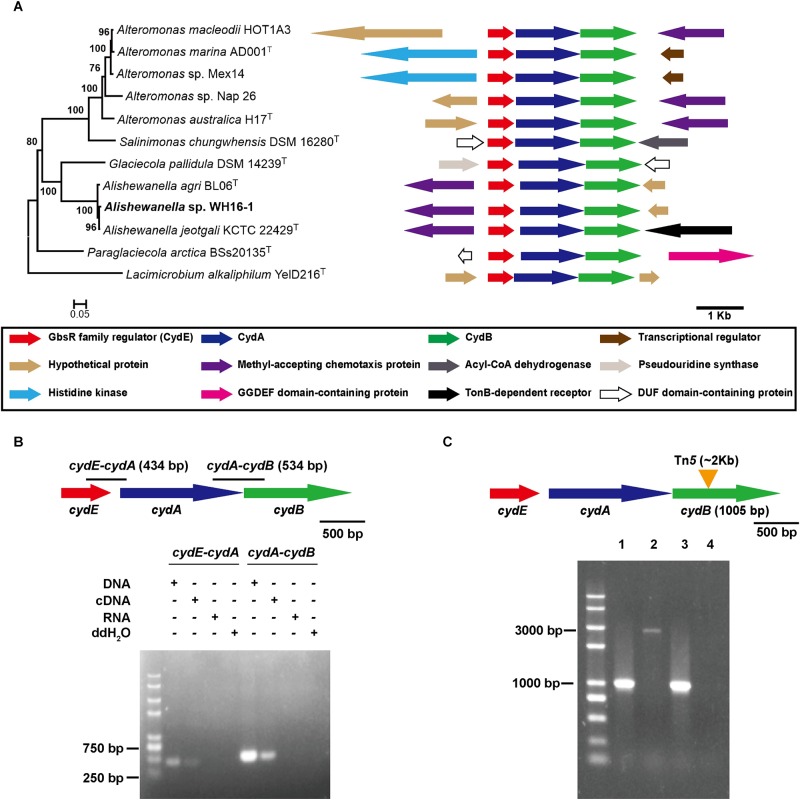
Analysis of *cytbd* gene operon, *cydB* mutation, and complementation. **(A)** The amino acid-based phylogenetic relationship of the *cytbd* gene cluster and its adjacent gene arrangement in Alteromonadaceae family members. **(B)** *cydE*, *cydA*, and *cydB* were co-transcribed. **(C)** Physical evidence of *cydB* mutation and complementation. Lanes 1–3 are the PCR-amplified products of wild-type, Δ*cytbd*, and Δ*cytbd*-C by primers *cydB*F/*cydB*R, respectively. Lane 4 is a negative control. The Tn*5* insert fragment is also approximately 1.8 kb. Consequently, the PCR product of the mutant strain is approximately 1.8 kb longer than PCR products of the wild-type and complemented strains.

The *cytbd* operon sequence is conserved in the *Alteromonadaceae* strains based on phylogenetic analysis (**Figure 1A**). Moreover, the gene arrangement is also similar in these strains (**Figure 1A**). The genes coding for CydE (AAY72_09270) and CydA (AAY72_09265) were identified adjacent to *cydB*. The operon was located in contig 1 of the genome sequence. To gain more insight, RT-PCR was carried out. The forward and inverse primers used for RT-PCR were designed to overlap each two adjacent genes. The results of RT-PCR showed that DNA fragments between the three genes (*cydE*/*cydA* and *cydA*/*cydB*) were amplified with DNA and cDNA templates. It implied that *cydE*, *cydA*, and *cydB* were co-transcribed in an operon (**Figure 1B**). To verify the function of Cytbd, a complementation experiment was carried out. The complete *cytbd* operon including *cydE*, *cydA*, and *cydB* was introduced into the mutant strain Δcyt*bd* and confirmed by PCR using primers *cydB*F/*cydB*R (**Figure 1C**) and DNA sequencing. This generated the complemented strain Δ*cytbd*-C.

### The Expression of Cytbd Was Induced by Sulfate

**FIGURE 2:**
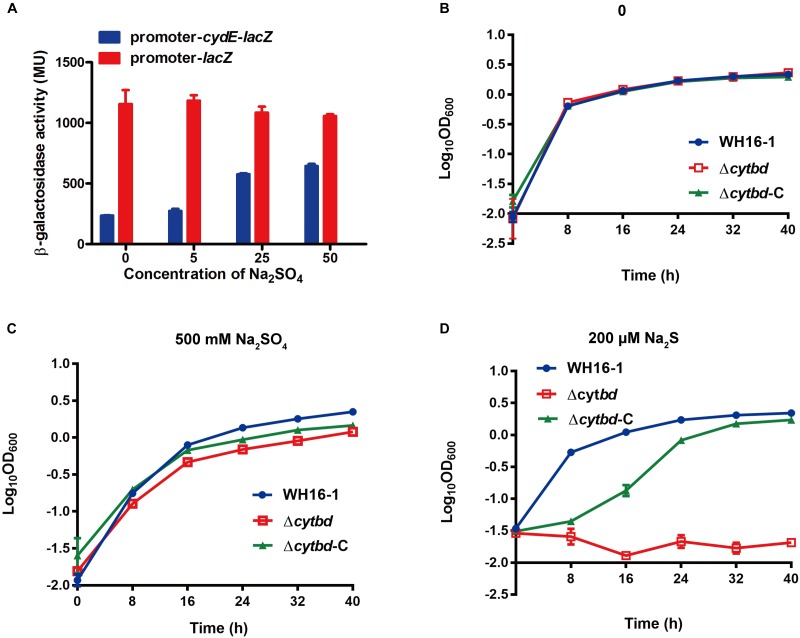
Cytbd is induced by sulfate and contributes to sulfate and sulfide resistance. **(A)** Sulfate effects on Cytbd expression. β-Galactosidase activities were increased after adding sulfate or removing *cydE* behind the promoter. The growth of WH16-1 (wild type), Δ*cytbd* (mutant strain), and Δ*cytbd*-C (complemented strain) with 0 **(B)**, 500 mM Na_2_SO_4_ **(C)**, or 200 μM Na_2_S **(D)** in LB medium. The values represent averages and standard deviations of three replicates.

*Escherichia coli* DH5α-pLSP*-*promoter-*lacZ* and *E. coli* DH5α- pLSP*-*promoter-*cydE*-*lacZ* were constructed (**Supplementary Figure S1**) to test the expression of Cytbd protein. When cells were incubated in LB medium for 4 h without sulfate, the β-galactosidase activity was higher without CydE, indicating that CydE repressed the activity of the *cydE* promoter (**Figure 2A**). Furthermore, the β-galactosidase activity of *E. coli* DH5α-pLSP*-*promoter-*cydE*-*lacZ* was upregulated when sulfate was added (**Figure 2A**). However, Cytbd was constitutively expressed when chromate or sulfide were added (data not shown).

### Cytbd Contributes to Sulfide and Sulfate Resistance

The wild-type, mutant, and complemented strains were used in sulfate- and sulfide-sensitivity tests. Cultures containing corresponding strains without sulfate and sulfide were used as controls (**Figure 2B**). The results showed that with the addition of 500 mM Na_2_SO_4_ or 200 μM Na_2_S, the wild-type strain grew almost as well as the ones without the addition of Na_2_SO_4_ or Na_2_S (**Figures 2C** vs. **B**). However, the growth of strain Δ*cytbd* was partially inhibited with the addition of sulfate (**Figure 2C**) and completely inhibited with the addition of sulfide (**Figure 2D**), and the complemented strains were partially recovered to the wild-type levels. These results indicated that Cytbd was weakly associated with sulfate resistance, but it was very essential for sulfide resistance in strain WH16-1. Sulfate appeared not to be very toxic to strain WH16-1 since the addition of 500 mM sulfate had almost no effect on its growth.

### Cytbd Contributes to Chromate Resistance and Reduction

**FIGURE 3:**
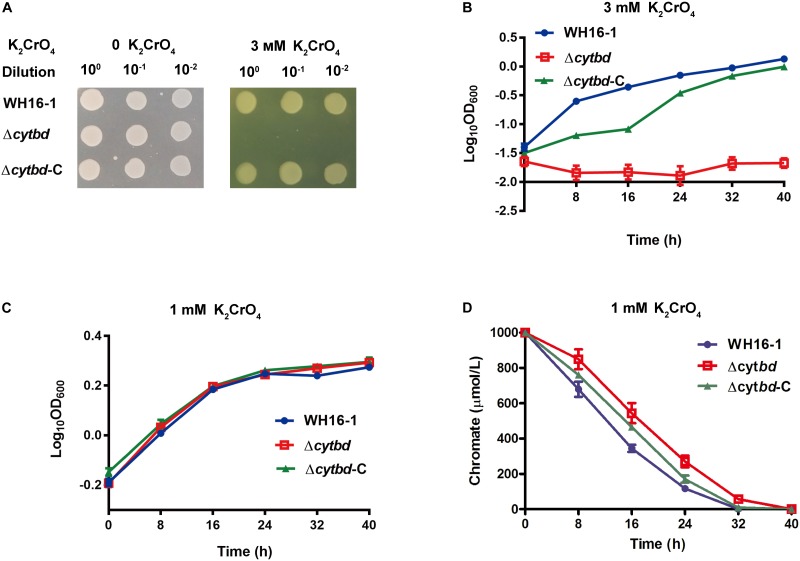
Effects of Cytbd on chromate resistance and reduction. **(A)** The growth of WH16-1 (wild type), Δ*cytbd* (mutant strain), and Δ*cytbd*-C (complemented strain) with 0 or 3 mM K_2_CrO_4_ on an LB medium plate. **(B)** The growth curve of WH16-1, Δ*cytbd*, and Δ*cytbd*-C with 3 mM K_2_CrO_4_ in LB medium. K_2_CrO_4_ was added at the beginning (**A** and **B**). The growth **(C)** and K_2_CrO_4_ reduction **(D)** curves of WH16-1, Δ*cytbd*, and Δ*cytbd*-C with 1 mM K_2_CrO_4_ in LB medium. To maintain similar growth conditions, K_2_CrO_4_ was added until OD_600_ reached 0.6 (**C** and **D**). Every sample was prepared in triplicate, and the results are presented as the mean values.

A chromate sensitivity test was also performed. The chromate sensitivity test was performed on LB plates and in LB medium. The results showed that the chromate resistance of the mutant strain was noticeably weaker than in the wild-type and the complemented strain (**Figures 3A,B**). The chromate minimal inhibition concentration (MIC) of the mutant strain was 3 mM, while for the wild type, it was 45 mM (Xia et al., 2016). In addition, the Cr(VI) reduction ability of the mutant strain was also somewhat weaker than the wild-type and complemented strain (**Figure 3D**) under similar growth conditions (**Figure 3C**).

### Cytbd Protects Against Cellular Oxidative Stress

To achieve a better understanding of how Cyt*bd* contributes to chromate resistance, a series of experiments were carried out. First, the membrane protein of the wild-type, mutant, and complemented strain was extracted and reacted with H_2_O_2_ and chromate. The H_2_O_2_ decomposition activity of the Δ*cytbd* membrane protein was noticeably lower than the wild type and Δ*cytbd-*C (**Supplementary Figure S2A**), while chromate reduction showed no significant difference (data not shown). Furthermore, the cytoplasmic H_2_O_2_ contents were measured to reflect the cellular oxidative stress. Without K_2_CrO_4_, there were no significant differences in H_2_O_2_ content among strains WH16-1, Δ*cytbd*, and Δ*cytbd-*C (**Supplementary Figure S2B**). When exposed to 1 mM K_2_CrO_4_, the H_2_O_2_ contents of all three strains were increased (**Supplementary Figure S2B**). However, the H_2_O_2_ content in the mutant strain was higher than that in the wild-type and the complemented strain with K_2_CrO_4_ (**Supplementary Figure S2B**). A similar result was obtained based on the inhibition zone test for H_2_O_2_ sensitivity. The diameter of the inhibition zone of the mutant strain was visibly larger than for the other two strains (**Supplementary Figure S2C**). This finding means that the mutant strain was more sensitive to H_2_O_2_. In addition, the MIC to H_2_O_2_ of the mutant strain was 0.5 mM, which was lower than that of the wild-type and the complemented strain (**Supplementary Figure S2D**).

Interestingly, the mutant strain lost partial capability for H_2_O_2_ decomposition. Hence, it was more sensitive to H_2_O_2_ compared to the wild-type and complemented strain. As a result, we inferred that the Cytbd catalyzes the reduction of cytoplasmic oxidative stress to enhance Cr(VI) resistance in strain WH16-1, but it does not directly catalyze chromate reduction.

### Sulfate Enhances Chromate Resistance Level and Reduction Efficiency

**FIGURE 4:**
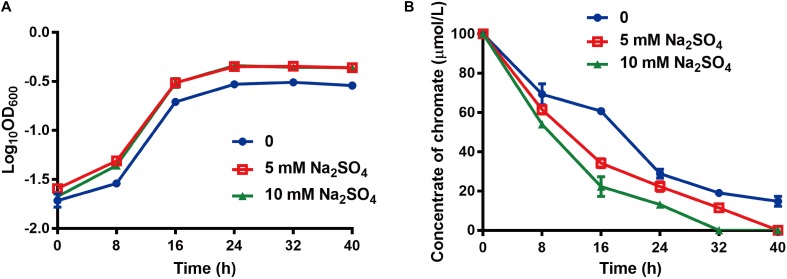
Effects of sulfate on chromate resistance and reduction. **(A)** The growth curve of strain WH16-1 with various amounts of additional sulfate in CDM medium. **(B)** The chromate reduction curve of strain WH16-1 with various additional Na_2_SO_4_. Data are shown as the mean of three biological replicates ± SD.

The effects of sulfate on chromate resistance and reduction were examined since sulfur metabolism is relevant to chromate metabolism in many bacteria (Viti et al., 2014), and the above results showed that sulfate and chromate resistance are both associated with Cytbd. The growth of strain WH16-1 showed no significant difference with or without additional Na_2_SO_4_ in the absence of added K_2_CrO_4_ (data not shown) but was affected after adding K_2_CrO_4_ (**Figure 4A**). However, growth was much better with the addition of Na_2_SO_4_ (**Figure 4A**). Additionally, the Cr(VI) reduction ability of strain WH16-1 increased with increasing concentrations of Na_2_SO_4_ (**Figure 4B**).

### Interaction Between Regulator CydE and the Promoter Region of *cytbd* Operon

**FIGURE 5:**
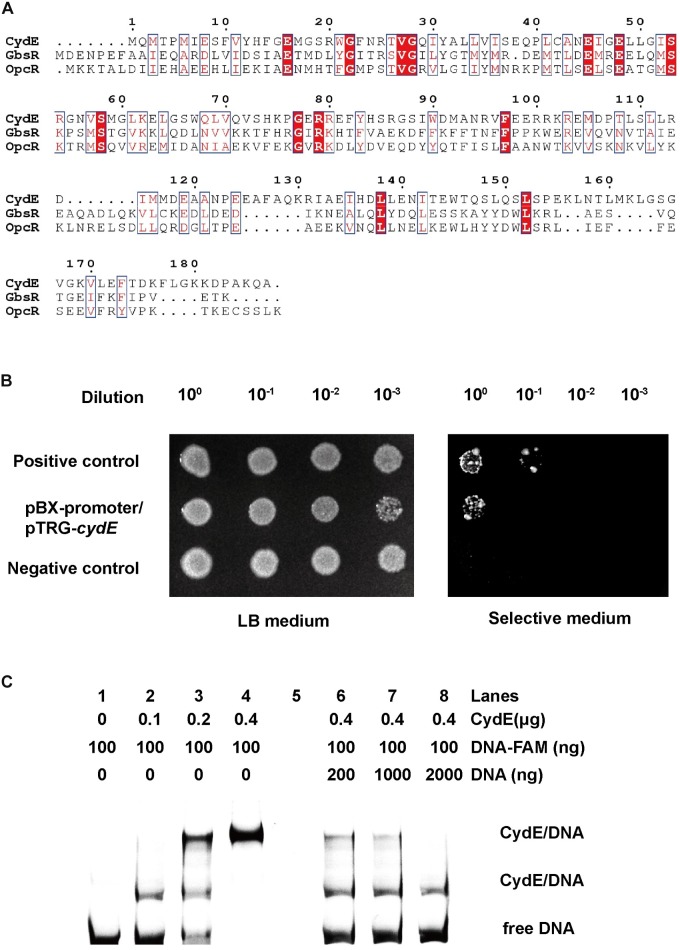
Sequence alignment of CydE and its interaction with the *cydE* promoter region. **(A)** Multiple sequence alignment was performed using ClustalW and Espript 3.0. The identical and elevated level of similarity residues are shown in red backgrounds and red boxes, respectively. The amino acid sequences of *Bacillus subtilis* GbsR (P71015) and *Bacillus subtilis*_OpcR (O34709) were selected from the UniProt database. **(B)** Bacterial one-hybrid assay. Co-transformants containing pBX-Mt2031p/pTRG-Rv3133c (Rv3133c protein can integrate with Mt2031p promoter) and empty vector pBXcmT/pTRG (without protein and promoter DNA) were used as positive and negative controls, respectively. Cells of positive and negative controls and the reporter strain containing plasmids pBX-promoter (promoter sequence of *cydE*) and pTRG-*cydE* (coding for CydE protein) were grown to an OD_600_ of 1.0, and 2 μL of each was spotted onto His-selective medium (+3AT, +Str^r^) and LB plates (–3AT, –Str^r^). **(C)** EMSA assay. Lanes 1–4, band shifts were enhanced by increased CydE. Lanes 6–8, FAM-labeled DNA was competed by unlabeled DNA. The result showed two migration bands. It indicated that there are two binding sites for the CydE in the *cydE* promoter region.

CydE is homologous to the GbsR-type regulator based on the results of BLASTP in NCBI. It shares 18.8% and 19.4% similarities with GbsR and OpcR, respectively. Next, we aligned CydE with the two reported GbsR regulators (**Figure 5A**). The results showed that CydE harbored the same conserved amino acids as the GbsR-type regulators. These conserved amino acid residues may be involved in DNA binding. GbsR-type regulators usually act as repressors of gene expression (Nau-Wagner et al., 2012; Lee et al., 2013).

To examine the regulation function of CydE, we first used a bacterial one-hybrid system to test the protein–DNA interaction based on the transcriptional activation of *HIS3* (imidazoleglycerol-phosphate dehydratase gene involved in histidine biosynthesis) and *aadA* (streptomycin resistance gene) (Guo et al., 2009). The promoter of *cydE* (**Supplementary Figure S1A**) was cloned into upstream of *HIS3–aadA* in the reporter vector pBXcmT, while the CydE coding region (**Supplementary Figure S1A**) was introduced into the pTRG vector. Both constructed vectors were then transferred into a histidine synthesis defective and streptomycin (Str) sensitive strain. The generated strain and positive control strain grew well on the screening plate (Guo et al., 2009) containing 3-amino-1,2,4-triazole (3-AT) and Str, while the negative control strain did not grow. The results demonstrated that CydE could interact with the promoter of the *cytbd* operon *in vivo* (**Figure 5B**).

Next, the purified His-tag CydE (**Supplementary Figure S3**) and *cydE* promoter DNA (**Supplementary Figure S1A**) were used to test the interaction *in vitro* using EMSA. With increasing amounts of CydE, the free DNA substrates gradually disappeared, while the intensity of the shifted DNA band increased (**Figure 5C**). Moreover, the unlabeled DNA substrate could competitively inhibit the binding of CydE to the labeled DNA substrate (**Figure 5C**).

**FIGURE 6:**
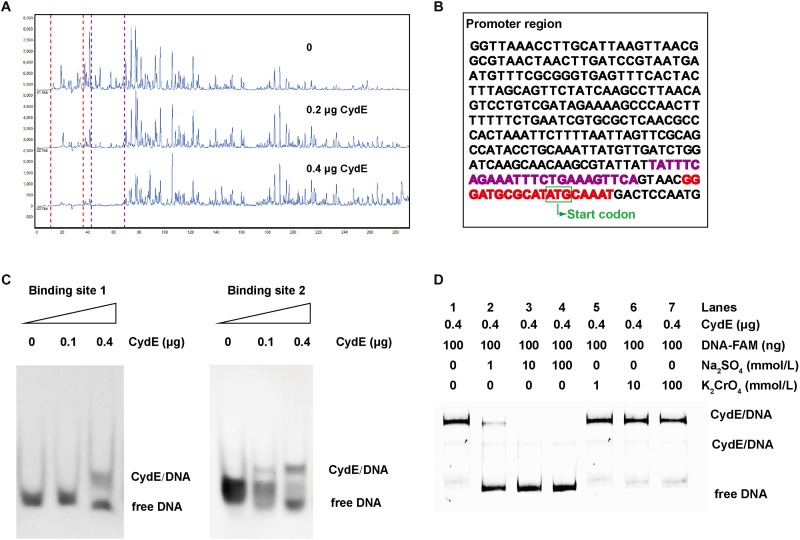
CydE binding sites and derepression analysis. **(A)** DNA footprinting. FAM-labeled DNA was the same as used in the EMSA and it was incubated with CydE in various amounts. Electropherograms indicated the protection pattern of DNA in different concentrations of CydE. **(B)** The promoter sequence of *cydE*. The red and purple highlighted sequences are binding sites 1 and 2, respectively. **(C)** EMSA for verification of the binding sites of CydE in the promoter region of *cydE*. The DNA of binding sites 1 and 2 sequences was synthesized and then incubated with CydE protein. The result showed that both the binding sites were shifted after adding CydE. **(D)** Derepression analysis. The free DNA band is shown with the addition of Na_2_SO_4_, while no similar phenomenon was observed with K_2_CrO_4_.

To identify the binding site of CydE, DNA footprinting was carried out. With increasing amounts of CydE, decreases in two sites of the peaks were observed (**Figure 6A**), indicating that there are two binding sites. This is consistent with the EMSA results. The sequences of the binding sites were TATTTCAGAAATTTCTGAAAGTTCA and GGGATGCGCATATGCAAAT (**Figure 6B**). The two binding site sequences were synthesized and then incubated with CydE. The EMSA result showed that both binding sites could interact with CydE (**Figure 6C**).

### CydE Is a Repressor and Can Be Derepressed by Sulfate

To investigate the repression ability of CydE, an EMSA derepression experiment was performed. The results showed that the free DNA increased when more sulfate was added (**Figure 6D**). However, the phenomenon was not observed when chromate was added (**Figure 6D**). These results are coincided with those of the *lacZ* reporter assay (**Figure 2A**). All these results demonstrated that CydE can repress the expression of the *cytbd* operon and sulfate addition results in derepression.

## Discussion

*Alishewanella* sp. WH16-1 is a sulfate- and chromate-reducing bacterium. According to our previous study, this strain can produce H_2_S during cultivation and has complete sulfate assimilation reduction pathway genes (*cysCDNHIJ*) (Xia et al., 2016; Zhou et al., 2016). It possesses high chromate resistance and reduction ability (Xia et al., 2016, 2018). In this study, we found that Cytbd was involved in sulfide and chromate resistance in *Alishewanella* sp. WH16-1. The function of Cytbd in sulfide resistance was previously reported in *E. coli* (Forte et al., 2016; Korshunov et al., 2016), where sulfide can inactivate heme–copper family cytochrome oxidase but not Cytbd (Forte et al., 2016; Korshunov et al., 2016). Under sulfide stress, Cytbd may play a key role in cell respiration. To our knowledge, this is the first report showing that Cytbd is associated with chromate resistance and reduction.

**FIGURE 7:**
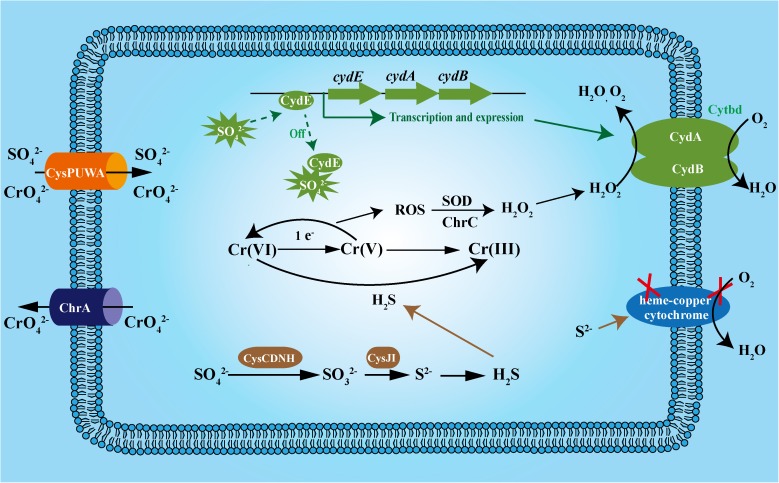
The proposed mechanism by which Cytbd contributes to sulfide and chromate resistance in *Alishewanella* sp. WH16-1. CydE, GbsR family regulator; CydA, Cytochrome *bd* complex subunit A; CydB, Cytochrome *bd* complex subunit B; ChrA, chromate efflux protein; CysPUWA, sulfate/chromate transporter; CysND, sulfate adenylyltransferases; CysC, adenylylsulphate kinase; CysH phosphoadenylylsulphate reductase; CysIJ, sulfite reductase.

In strain WH16-1, Cytbd is induced by sulfate and is essential for decomposing H_2_O_2_ to reduce cellular oxidative stress. In addition, Cytbd contributed to sulfide resistance, and sulfide can be used as a reductant to reduce chromate. These findings explain why Cytbd is important in coupling with chromate stress and the chromate resistance mechanism of strain WH16-1 appears to be indirect (**Figure 7**). Cytbd also plays an important role in resistance to other environmental stresses such as low oxygen, nitrosative, and oxidative stresses, since Cytbd can use O_2_, NO, and H_2_O_2_ as electron acceptors (Borisov et al., 2013; Giuffre et al., 2014; Roop et al., 2015). The electron transformation models between Cytbd and O_2_/NO have been clarified (Giuffre et al., 2014). Under low oxygen conditions, Cytbd is regulated by Arc, Fnr, or CydR in *E. coli* (Borisov et al., 2011) and *A. vinelandii* (Wu et al., 1997).

Another important achievement of this study is the finding of a novel regulation mechanism of Cytbd transcription. Previously, a GbsR-family protein was reported as an intracellular choline sensor (Nau-Wagner et al., 2012; Lee et al., 2013). In this study, we identified a GbsR-family protein, CydE, which is Cytbd’s repressor and it is inactivated by high sulfate concentration. In this way, high amount of sulfate can stimulate Cytbd transcription. Thus, sulfate could enhance chromate resistance in *Alishewanella* sp. WH16-1 (**Figure 7**). We speculate that numerous factors including the following may cause such enhancement. (i) Sulfate assimilation products such as S^2-^, Cys, and GSH can directly reduce chromate (Thatoi et al., 2014; Joutey et al., 2015; Qian et al., 2016). (ii) S^2-^ can be used for Fe–S cluster synthesis. A potential Cr(VI) reductase (4Fe–4S ferredoxin, AAY72_06850) was also identified by the Tn*5* transposon mutagenesis in this study. Previously, ferredoxin and hydrogenase, which contain the Fe–S cluster as the active group, were reported to be associated with chromate reduction (Chardin et al., 2003). In addition, proteins associated with Fe–S cluster biogenesis, such as IscRs, are involved in multiple stress responses (Liu et al., 2015; Romsang et al., 2015). (iii) Sulfate induces the expression of Cytbd, and Cytbd is essential for chromate resistance and reduction.

On the other hand, sulfate was reported to have no effect on Cr(VI) reduction in some bacteria (Shen and Wang, 1994; Campos et al., 1995; Liu et al., 2006) or even inhibited Cr(VI) reduction in some cases (Wang, 2000; Çetin et al., 2008). The different phenomena reflect various Cr(VI) reduction mechanisms of bacteria. Some bacteria cannot reduce sulfate to produce H_2_S and do not use sulfate and chromate as terminal electron acceptors (Liu et al., 2006). Accordingly, sulfate has no noticeable effect on chromate reduction in these bacteria. Other bacteria use chromate as a terminal electron acceptor under anaerobic conditions (Wang, 2000; Liu et al., 2006). In some cases, sulfate could inhibit the activity of chromate reductase competitively (Park et al., 2000) and consequently inhibit chromate reduction in these microorganisms. These reports and our results suggest that the chromate detoxification mechanisms of selected bacteria are quite varied.

## Conclusion

We showed that Cytbd contributes to chromate resistance, which can be explained by the ability of Cytbd to catalyze the decomposition of H_2_O_2_ to protect against H_2_O_2_-related oxidative stresses. Furthermore, Cytbd contributes to the resistance of sulfide, and sulfide could act as a reductant to reduce chromate. In addition, Cytbd’s expression is negatively regulated by the GbsR family regulator CydE and derepressed by sulfate. Hence, sulfate could enhance chromate resistance and reduction in *Alishewanella* sp. WH16-1.

## Author Contributions

XX designed, analyzed and interpreted the experiments, and prepared the manuscript. SW participated in the *lacZ* report gene and sulfate-/sulfide-sensitive experiments. LL and BX participated in the Tn*5* transposon test. GW designed the study and revised the draft manuscript.

## Conflict of Interest Statement

The authors declare that the research was conducted in the absence of any commercial or financial relationships that could be construed as a potential conflict of interest.
