# Supplementary material for: The Cytochrome bd Complex Is Essential for Chromate and Sulfide Resistance and Is Regulated by a GbsR-Type Regulator, CydE, in Alishewanella Sp. WH16-1
Source: Front Microbiol. 2018 Aug 10;9:1849. doi: 10.3389/fmicb.2018.01849 (PMC6096048; doi:10.3389/fmicb.2018.01849)
Supplement: Supplementary file 1 [file Data_Sheet_1.PDF]

The Cytochrome *bd* complex is essential for sulfide, sulfate and chromate resistance and is regulated by a GbsR-type regulator, CydE, in *Alishewanella* sp. WH16-1

Xian Xia, Shijuan Wu, Liqiong Li, Biao Xu, Gejiao Wang*

State Key Laboratory of Agricultural Microbiology, College of Life Sciences and Technology, Huazhong Agricultural University, Wuhan, 430070, P. R. China

TABLE S1 Bacterial strains and plasmids used in this study

Strains or plasmids	Relevant characteristic(s)	Source or reference
**Bacterial strains**		
***Alishewanella* sp.**		
WH16-1	Wild type; Cr(VI)^r; Rif^r, Km^s, Tc^s , Cm^s	This study
△cyt*bd*	Mutant of WH16-1(Rif); Tn5 inserted in *cydB*; Rif^r, Km^r	This study
△cyt*bd*-C	Mutant of WH16-1(Rif) complemented with the Cytbd oxidase operon cloned in pCT-Zori vector; Rif^r, Km^r, Cm^r	This study
Escherichia coli		
E. coli S17-1(λ*pir*)	*Tpr Smr recA thi pro hsdR$^-$ hsdM$^+$*. RP4:2Tc:Mu:Km T7, λ*pir*	(Chen et al., 2015a)
E. coli DH5α	F$^-$ *endA1 hsdR17 supE44 thi-1 recA1 deoR gyrA96 relA1* Δ (*argF-lacZYA*)U169 Φ80d*lacZ*M15 λ$^-$	Promega
XL1-Blue	Δ(mcrA)183Δ(mcrCB-hsdSMR-mrr)173 endA1 supE44 thi-1 recA1 gyrA96 relA1lac [F' proAB lacIqZΔM15 Tn*5* Km^r]	Stratagene
BL21	B F– *dcm ompT hsdS* ($r_B^-m_B^-$) *gal* λ(DE3)	Novagen
**Plasmids**		
pRL27	Tn*5*-based transposon vector, *ori*R6K , Km^r	(Larsen et al., 2002)
pCT-Zori	Cm^r	(Chen et al., 2015a)
pCT-Zori-*cytbd*	pCT-Zori vector cloned with the whole *cytbd* operon	This study
pLSP	*lacZ* reporter gene vector	(Li et al., 2015)
pLSP-Promoter	pLSP containing promoter region of *cytbd* operon	This study
pLSP-Promoter-*cydE*	pLSP containing promoter and *cydE* coding region of *cytbd* operon	This study
pTRG	Tet^r, for bacterial one-hybrid assay	Stratagene
pTRG-*cydE*	pTRG containing *cydE* coding region	This study
pBXcmT	Cm^r, for bacterial one-hybrid assay	(Guo et al., 2009)
pBXcmT-Promoter	pBXcmT containing *cytbd* operon promoter region	This study
pET28a	His6 Tag expression vector, Km^r	Novagen
pET28a-*cydE*	*cydE* in frame fusion to the multiple sites of pET-28a	This study

TABLE S2 Primers used in this study

Primer	Sequence(5'-3')	Description
tpnRL17-1	AACAAGCCAGGGATGTAACG	Used for plasmid rescue
tpnRL13-2	CAGCAACACCTTCTTCACGA	
*cydE-cydA*F	TAGCCGCGGTAGTATCTG	Used to amplify *cydE-cydA*
*cydE-cydA*R	GATGGTAATGGTTGGGAA	
*cydA-cydB*F	GGTGTAGCCTGCGGTTGT	Used to amplify *cydA-cydB*
*cydA-cydB*R	TCCCAGAACGGACCAATAG	
*cytbd*F	AAAGAGCTCGGTTAAACCTTGCATTAAGT	Used for complementation of WH16-1-*cydB*(Tn*5*)
*cytbd*R	AAAAAGCTTTCTCATTGTCTTATTAGTAATACTT	
LacPF	AAAGAATTCGGTTAAACCTTGCATTAAGT	Used for construction the *lacZ* reporter gene verctor
LacPR	AAAGGATCCCATTGGAGTCATTTGCATA	
LacP-*cydE*R	AAAGGATCCACAGGTGGCAAAAACAG	
*cydE*F	AAAGGATCCATGCAAATGACTCCAATG	Used for construction CydE over-expression vector with pET28a
*cydE*R	AAAAAGCTTGGCTTGTTTTGCCGGATC	
BohpF	AAAGGATCCATGCAAATGACTCCAATG	Used for bacterial one-hybrid experiment of *cytbd* cluster regulatory region DNA
BohpR	AAAGAATTCAGAACAACAGGTGGCAAA	
BohsF	AAAGGATCCATGCAAATGACTCCAATG	Used for construction of CydE protein expression vector with pTRG
BohsR	AAAGAATTCAGAACAACAGGTGGCAAA	
PromoterF	GGTTAAACCTTGCATTAAGT	Used to amplify promoter region sequence of *cytbd* cluster
PrompterR	CATTGGAGTCATTTGCATA	
FAM-PrompterR	FAM-CATTGGAGTCATTTGCATA	Used to amplify FAM labeled promoter region of *cytbd* operon

* The underlined sequence denotes the restriction enzyme sites.

A

GGTTAAACCTTGCATTAAGTTAACGGCGTAACTAACTTGATCCGTAATGAATGTTTCGCGGGTGAGTTTCAC
TACTTTAGCAGTTCTATCAAGCCTTAACAGTCCTGTCGATAGAAAAGCCCAACTTTTTTTCTGAATCGTGCGC
TCAACGCCCACTAAATTCTTTTAATTAGTTCGCAGCCATACCTGCAAATTATGTTGATCTGGATCAAGCAACA
AGCGTATTATTATTTCAGAAATTTCTGAAAGTTCAGTAACGGGATGCGCATATGCAAATGACTCCAATGATTG
AATCCTTTGTTTACCACTTTGGCGAAATGGGCAGCCGTTGGGGCTTTAACCGTACCGTTGGCCAAATTTACG
CCCTGCTGGTGATTAGCGAGCAACCGCTCTGTGCCAATGAAATTGGTGAGCTGCTGGGGATTTCGCGCGG
CAATGTCAGTATGGGGTTAAAAGAGTTGGGTAGCTGGCAGCTGGTGCAGGTTAGCCATAAACCCGGCGAG
CGGCGCGAGTTTTATCATAGCCGCGGTAGTATCTGGGATATGGCCAACCGGGTGTTTGAAGAGCGGCGCA
AACGGGAAATGGACCCGACGCTTAGCTTACTGCGTGACATTATGATGGACGAAGCGGCTAATCCGGAAGA
AGCCTTTGCCCAAAAGCGCATCGCCGAAATTCACGATCTGCTGGAGAACATTACTGAATGGACGCAAAGCC
TGCAATCCTTGTCGCCGGAGAAGCTGAATACCTTAATGAAGTTAGGCTCCGGCGTTGGCAAGGTACTGGAA
TTTACCGATAAGTTTTTAGGTAAAAAAGATCCGGCAAAACAAGCCTAGCTGTTTTTGCCACCTGT

B **C**

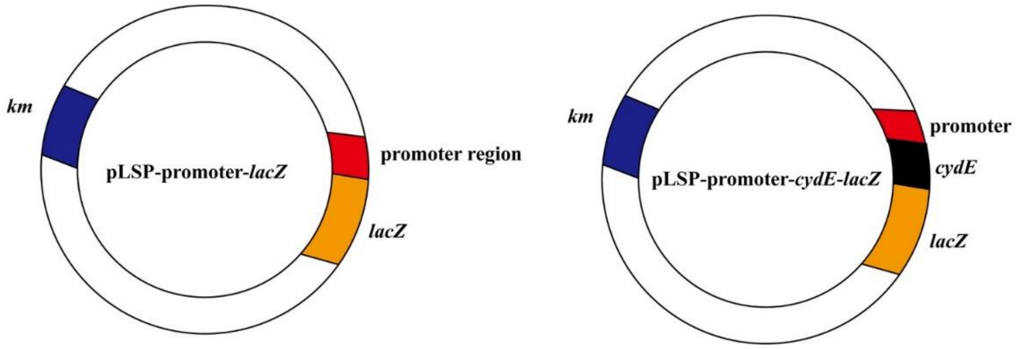

FIG S1 Sequence of *cydE* and its promoter and the construction of related vectors. (A) The red nucleotides are the *cydE* promoter region and 18 nucleotides of *cydE*, and the underlined nucleotides are *cydE* coding sequence; (B) The *cydE* promoter sequence (red) was cloned into the pLSP-kt2*lacZ* plasmid to generate the pLSP-promoter-*lacZ* vector; (C) All of the sequence shown in "A" including *cydE* promoter and *cydE* was cloned into the pLSP-kt2*lacZ* plasmid to generate the pLSP-promoter-*cydE*-*lacZ* vector. For the construction of the pBX-promoter vector in the bacterial one-hybrid assay (FIG 5), the promoter sequence of *cydE* (red) was cloned into the pBXcmT plasmid.

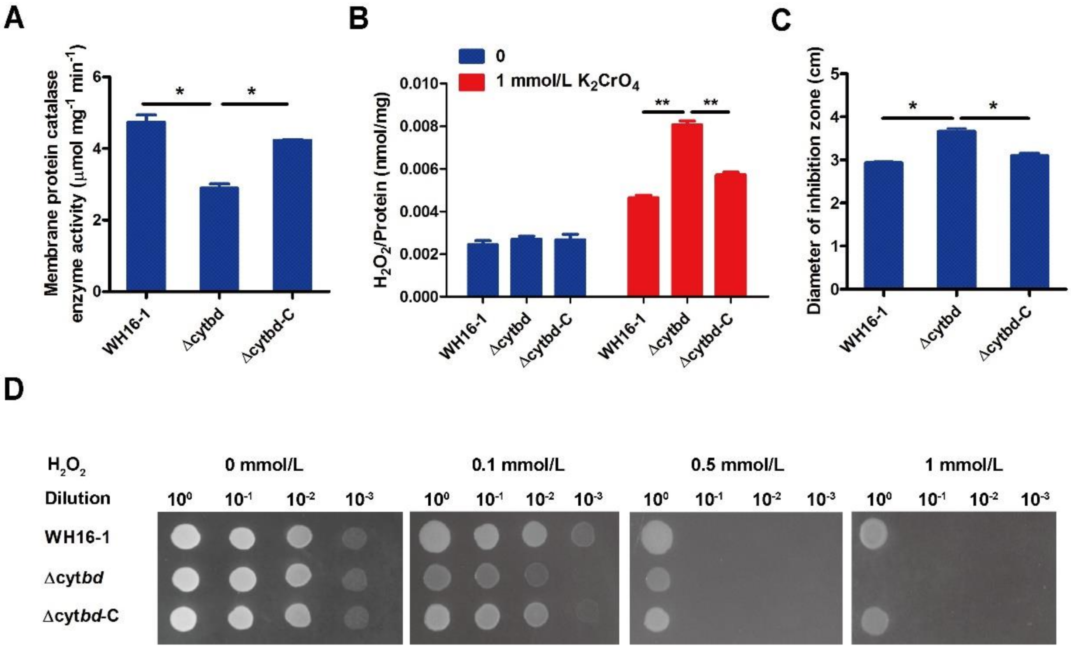

FIG S2 Effects of Cytbd on the reduction of cellular oxidative stress in strain WH16-1, mutant Δ*cytbd* and complementary strain Δ*cytbd*-C. (A) The H_2O_2 decomposition ability of membrane proteins. (B) Cellular H_2O_2 contents with 0 and 1 mmol/L K_2CrO_4, respectively. (C) The inhibition zones when exposed to 3% H_2O_2. (D) H_2O_2 sensitive tests on LB plates. Every sample was prepared in triplicates and the results are presented as mean values. *, remarkable difference; **, extremely remarkable difference.

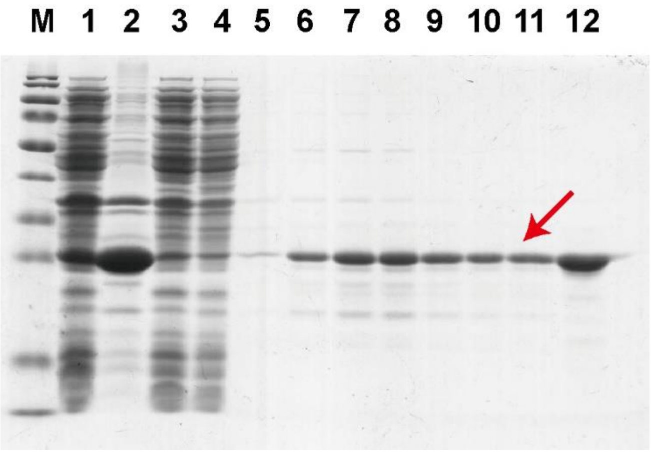

FIG S3 Purification of CydE. Lanes 1 and 2 show cell lysis product suspensions and precipitates after centrifuging, respectively. Lanes 3-5 and 6-12 show proteins eluted from a nickel-ion affinity chromatography column by 200 and 500 mM imidazole, respectively. The red arrow marks proteins that were used in a subsequent EMSA test.
